# Multivariate analysis of prognostic factors in patients with pulmonary actinomycosis

**DOI:** 10.1186/1471-2334-14-10

**Published:** 2014-01-09

**Authors:** Ji Young Park, Taehoon Lee, Hongyeul Lee, Hyo-Jeong Lim, Jinwoo Lee, Jong Sun Park, Young-Jae Cho, Young Sik Park, Chang-Hoon Lee, Sang-Min Lee, Ho Il Yoon, Jae-Joon Yim, Chul-Gyu Yoo, Young Whan Kim, Sung Koo Han, Choon-Taek Lee, Jae Ho Lee

**Affiliations:** 1 Division of Pulmonary and Critical Care Medicine, Department of Internal Medicine, Seoul National University Bundang Hospital, Seoul National University College of Medicine, Seongnam, Korea; 2 Division of Pulmonary and Critical Care Medicine, Department of Internal Medicine and Lung Institute, Seoul National University College of Medicine, Seoul, Korea; 3 Department of Internal Medicine, Seoul National University Bundang Hospital, 166 Gumi-ro, Bundang-gu, Seongnam-si, Gyeonggi-do 463-707, the Republic of Korea

**Keywords:** Actinomycosis, Anti-bacterial agents, Treatment outcome, Prognostic factors, Pulmonary

## Abstract

**Background:**

There have been few studies of pulmonary actinomycosis, which is an uncommon anaerobic infection. Consequently, the optimal therapeutic regimen, appropriate duration of treatment, long-term prognosis, and factors predicting prognosis are not well established.

**Methods:**

We retrospectively reviewed the medical records of histopathologically confirmed cases of pulmonary actinomycosis seen between November 2003 and December 2012.

**Results:**

The study included 68 patients with a mean age of 58.4 ± 11.6 years. Of the 68, initial surgery was performed in 15 patients (22.1%), while the remaining 53 (77.9%) received antibiotic therapy initially. In the initial antibiotic group, 45/53 (84.9%) were cured without relapse (median antibiotic duration 5.3 months). 5/53 (9.4%) patients were refractory medically (median antibiotic duration 9.7 months), and 3/53 (5.7%) experienced a recurrence (median time to relapse 35.3 months). In the initial surgery group, 14/15 (93.3%) were cured and treatment failure occurred in one (6.7%). In the multivariate analysis, the absence of an antibiotic response at 1 month was the only independent factor associated with a poor treatment outcome, with an adjusted odds ratio of 49.2 (95% CI, 3.34–724.30). There was no significant difference in treatment outcome based on the size of the parenchymal lesion, comorbidities, whether intravenous antibiotics were used, antibiotic therapy duration, or whether the initial treatment was surgical.

**Conclusions:**

Antibiotic treatment with or without surgery was effective for treatment of pulmonary actinomycosis. Nevertheless, treatment failure or recurrence occurred in a considerable proportion of patients, especially those resistant to the initial antibiotic treatment.

## Background

Actinomycosis is a chronic, slowly progressive granulomatous disease caused by filamentous Gram-positive anaerobic or microaerophilic bacteria of the family Actinomycetaceae (genus *Actinomyces*) [1]. These normally colonize the mouth, colon, and vagina [2]. This can lead to infection at virtually any site in the body; pulmonary actinomycosis accounts for 15% of all actinomycosis cases [1,3]. Infection is thought to result from the inhalation or aspiration of oropharyngeal or upper gastrointestinal materials [1,4].

Pulmonary actinomycosis is difficult to diagnose and needs prolonged antibiotic treatment [1]. The diagnosis is suspected on admission in only 7–18% of patients who later turn out to have an actinomycosis infection [5,6]. Traditionally, patients with pulmonary actinomycosis are treated with high-dose intravenous (IV) penicillin at a dose of 18–24 million units daily for 2–6 weeks, followed by oral penicillin or amoxicillin for 6–12 months [2]. The principles of intensive, prolonged therapy were recommended in the late 1950s based on clinical experience [7,8]. However, the evidence for this recommendation is weak. Furthermore, several investigators have successfully treated extensive pulmonary actinomycosis with relatively brief courses of antibiotics [9,10]. Conversely, a recent study showed that medical treatment failure can occur in patients receiving appropriate antibiotics for pulmonary actinomycosis [6]. This is incompatible with the traditional concept of a medically treatable disease with a good prognosis [5]. However, no randomized controlled trial or large-scale retrospective study has compared antibiotic regimens or treatment modalities.

In this retrospective study, we investigated the long-term prognosis of pulmonary actinomycosis, evaluated the efficacy of specific antibiotics, and identified the clinical parameters predicting the treatment outcome.

## Methods

### Study population

We reviewed retrospectively the medical records of all patients with pulmonary actinomycosis seen at Seoul National University Bundang Hospital and Seoul National University Hospital between November 2003 and December 2012. This study was approved by the institutional review board of Seoul National University Bundang Hospital (B-1210-176-114).

### Study design and data acquisition

We selected patients whose actinomycosis lesions were confirmed by biopsy, using either surgical or nonsurgical methods. We collected clinical data on the patients, including smoking status, alcohol consumption, education status, underlying systemic disease, pulmonary comorbidity, diagnostic methods, and treatment modalities. We also reviewed the initial chest computed tomography (CT) images, which were available for all patients. The last follow-up day was defined as the day on which the last chest radiograph was conducted in the study hospitals.

### Definition and classification of treatment outcomes

Actinomycosis was confirmed by biopsy findings of Gram-positive branching filamentous organisms on Gram (or Gomori methenamine silver) staining or sulfur granules seen upon hematoxylin-eosin staining. Treatment outcomes were defined as cure (complete resolution of the main lesion after antibiotic treatment) or failure (persistence or progression of the lesion or uncontrolled symptoms despite proper antibiotics therapy). If patients were diagnosed with pathological or radiological evidence of relapsed pulmonary actinomycosis after being cured, they were classified as recurrent cases. Based on this classification, the treatment outcome was further categorized into good (cured) or poor (failure or recurrence) to identify predictors of treatment outcome.

### Statistical analysis

The duration of antibiotic treatment is expressed as the median and range. Categorical variables were analyzed using Pearson’s chi-square test or Fisher’s exact test. Continuous variables were analyzed using Student’s t-test or Mann–Whitney U-test. All variables were assessed in the univariate analyses. Age, sex, and variables showing a significant correlation (p ≤ 0.1) were included in the multivariate logistic regression analysis. A p value < 0.05 was considered to indicate statistical significance. All analyses were performed using SPSS for Windows ver. 18.0 (SPSS, Chicago, IL, USA).

## Results

### Baseline characteristics of the subjects

The baseline characteristics of the 68 patients are summarized in Table 1. The mean ± SD age was 58.4 ± 11.6 years, and 47 (69.1%) were male. Forty-two (65.6%) patients were current or ex-smokers. Nine patients were heavy drinkers who consumed alcohol more than four times per week. None of the 68 patients tested positive for human immunodeficiency virus. No patient had used long-term systemic corticosteroids. The most common symptoms were cough (n = 62), followed by sputum production (n = 54) and hemoptysis (n = 40). Massive hemoptysis defined as > 200 mL/day occurred in 9 (13.2%) patients. Diagnostic procedures were a non-surgical biopsy in 49 (72.1%) patients, CT-guided transthoracic needle aspiration or biopsy in 39 (57.4%), bronchoscopic biopsy in nine (13.2%), and endobronchial ultrasonography biopsy in one patient.

**Table 1:** Clinical characteristics of 68 patients with pulmonary actinomycosis

	**Number of observed (%)**	**Number of reported**
Mean age, years	58.4 ± 11.6	68
Sex, male (%)	47 (69.1)	68
Smoking		64
Never smoker	22 (34.4)	
Current or ex-smoker	42 (65.6)	
Alcohol drinking		61
Never drinker	32 (52.4)	
Social drinker	20 (32.8)	
Heavy drinker (>4 times/week)	9 (14.8)	
Systemic disease		68
Diabetes mellitus	11 (16.2)	
Malignancy	8 (11.8)	
Chronic liver disease	4 (5.9)	
Cerebral vascular accident	2 (2.9)	
Chronic kidney disease	1 (1.5)	
Long term steroid use	0 (0.0)	
Pulmonary comorbidities		68
COPD	13 (19.1)	
Tuberculosis	7 (10.3)	
Bronchiectasis	6 (8.8)	
Lung cancer	2 (2.9)	
Asthma	1 (1.5)	
Educational background		51
Low (elementary, middle school)	14 (27.4)	
Moderate (high school)	16 (31.4)	
High (college)	21 (41.2)	
Symptoms and signs		68
Cough	62 (91.2)	
Sputum production	54 (79.4)	
Hemoptysis	40 (58.8)	
Massive hemoptysis	9 (13.2)	
Chest pain	16 (23.5)	
Fever	16 (23.5)	
Weight-loss	16 (23.5)	
Dyspnea	15 (22.1)	
Chest CT findings		68
Consolidation	63 (92.6)	
Central necrosis	42 (61.8)	
Cavitation	13 (19.1)	
Peripheral subpleural lesion	44 (64.7)	
Pleural thickening	47 (69.1)	
Focal pleural effusion	19 (27.9)	
Mediastinal lymph node enlargement	27 (39.7)	
Size of parenchymal lesion, mean (cm, range)	3.9 (1.5-9.0)	

### Clinical course

**Figure 1:**
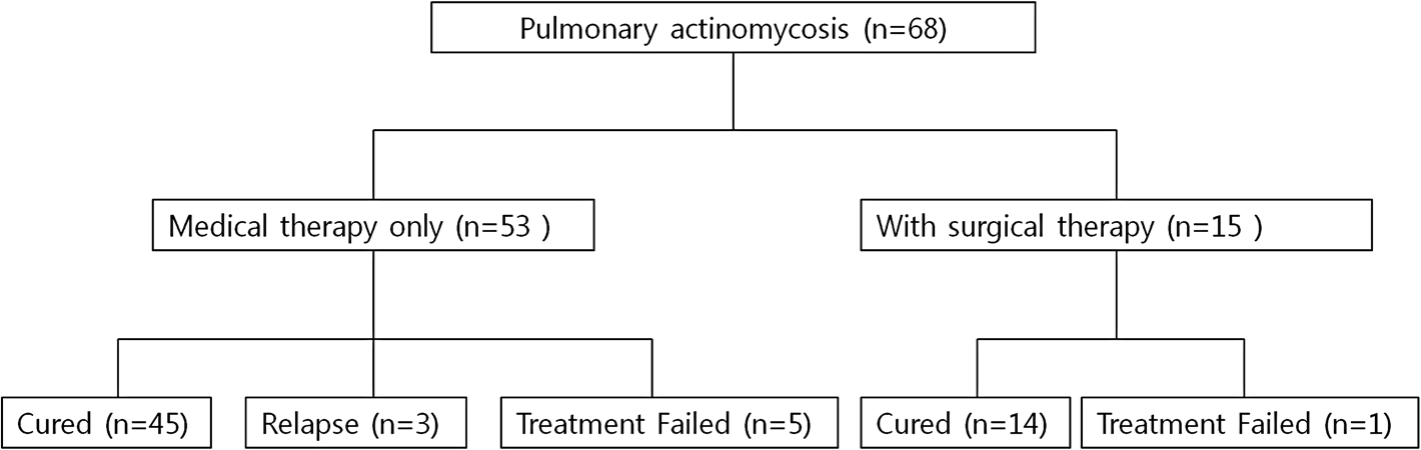
Schematic of the clinical course in the patients with pulmonary actinomycosis.

The mean follow-up duration was 37.7 (range 3.0–113.8) months. Initially, 53/68 (77.9%) patients were treated only with antibiotics and the other 15/68 (22.1%) patients underwent surgical treatment followed by antibiotics (Figure 1). Of the 53 patients who initially received antibiotic therapy, 45 (84.9%) were cured without relapse (median antibiotic duration 5.3 months). Five of the 53 (9.4%) patients were medically refractory (median antibiotic duration 9.7 months) and three of 53 (5.7%) patients experienced a recurrence (median time to relapse 35.3 months, range 20.1–42.6). All of the five medically refractory patients underwent rescue surgical treatment and received antibiotics after surgery. Their treatment was successful without complications. Of the patients with recurrence, two underwent surgical treatment and the other was treated using the same antibiotics as had been used successfully for the previous episode. With initial surgical treatment, cure occurred in 14/15 (93.3%) and treatment failure in 1/15 (6.7%). This surgical failure had used long-term antibiotics but did not respond well, and a second operation was planned. Fourteen of the 15 patients received oral antibiotic therapy after surgery (median 5.1 (range 0.4–15.7) months). One patient did not receive antibiotics after surgery (not relapsed). There were no differences in treatment outcomes regarding with or without initial surgical treatment (Table 2).

**Table 2:** Outcomes of pulmonary actinomycosis after treatment with antibiotics alone or with initial surgery followed by antibiotics

	**Initial antibiotics treatment**	**Initial surgical treatment**	**Total**	**P value**
	**(n = 53)**	**(n = 15)**	**(n = 68)**	
Treatment outcome				*P* = 1.000
Cured	45 (84.9)	14 (93.3)	59 (86.8)	
Failure	5 (9.4)	1 (6.7)	6 (8.8)	
Relapse	3 (5.7)	0 (0.0)	3 (4.4)	
Number of patients treated with IV antibiotics	23 (43.4)	15 (100.0)	38 (55.9)	*P* < 0.001
Duration of antibiotics				
IV antibiotics, day	6 (2–19)	8 (3–21)	7 (2–21)	*P* = 0.022
Oral antibiotics, month	5.4 (1.2-14.0)	5.1 (0.4-15.7)	5.3 (0.4-15.7)	*P* = 0.363

### Intravenous antibiotics

Thirty-eight (55.9%) patients initially received IV antibiotic treatment. The median duration was 7 (range 2–21) days. Various IV antibiotics were used, including third-generation cephalosporins (ceftriaxone, cefotaxime, cefpiramide) (n = 12), aminopenicillin with or without a β-lactamase inhibitor (ampicillin, amoxicillin) (n = 10), oxacephem (flomoxef) (n = 5), first-generation cephalosporins (cefazolin) (n = 4), penicillin (penicillin G) (n = 3), quinolone (levofloxacin, moxifloxacin) (n = 3), and clindamycin (n = 1). Among the five patients who failed initial antibiotics, four (80%) did receive IV antibiotics. Nevertheless, the proportion of patients who did not use IV antibiotics was not significantly different between cured and treatment-failed patients in the antibiotics-alone group (54% vs. 80%, *p* = 0.374).

### A comparison of treatment outcome with various oral antibiotics

The oral antibiotic regimens and outcomes of patients with pulmonary actinomycosis are shown in Table 3. Patients received oral antibiotics either initially or after IV antibiotics. Most patients received an oral beta-lactam with a beta-lactamase inhibitor (n = 55): amoxicillin clavulanate (AMX-CLV) in 30 patients and ampicillin sulbactam (AMP-SUL) in 25. Treatment failure occurred in 3/30 (10%) patients receiving AMX-CLV and 2/25 (8%) receiving AMP-SUL, with 3/25 (12%) relapses in the latter group. The AMX-CLV group had a higher cure rate than the AMP-SUL group, but the difference was not significant (90% vs. 80%, *p* = 0.295).

**Table 3:** Outcomes of treatment of pulmonary actinomycosis with various oral antibiotics

	**All patients (n = 68)**				**Initial antibiotics alone (n = 53)**			
	**Cured**	**Failure**	**Relapse**	**Total**	**Cured**	**Failure**	**Relapse**	**Total**
AMX-CLV	27 (90.0)	3 (10.0)	0 (0)	30	25 (89.3)	3 (10.7)	0 (0.0)	28
AMP-SUL	20 (80.0)	2 (8.0)	3 (12.0)	25	10 (71.5)	1 (7.1)	3 (21.4)	14
Amoxicillin	4 (80.0)	1 (20.0)	0 (0)	5	3 (75.0)	1 (25.0)	0 (0)	4
Ampicillin	2 (100)	0 (0)	0 (0)	2	2 (100)	0 (0)	0 (0)	2
Clindamycin	3 (100)	0 (0)	0 (0)	3	2 (100)	0 (0)	0 (0)	2
Others	3 (100)	0 (0)	0 (0)	3	3 (100)	0 (0)	0 (0)	3

AMX-CLV; Amoxicillin clavulanate.

AMP-SUL; Ampicillin sulbactam.

### Comparison of patients with good and poor outcomes

A good outcome was achieved in 59/68 (86.8%) of the cases and a poor outcome in 9/68 (13.2%), consisting of treatment failed (8.8%) and relapse (4.4%) (Table 4). The mean age of the poor outcome group was younger than that of the good outcome group (51.0 vs. 59.6 years; *p* = 0.038). The 1-month antibiotic response rate was significantly higher in the good outcome group than in the poor outcome group (92.7% *vs*. 22.2%; *p* < 0.001). There was no significant difference regarding symptoms, size of the parenchymal lesion, pulmonary comorbidities, systemic disease, with or without of IV antibiotics, or initial surgery. In the multivariate analysis, absence of a 1-month antibiotic response was an independent factor associated with a poor outcome, with an adjusted odds ratio of 49.2 (95% confidence interval (CI) 3.34–724.30). Age, sex, and the presence of hemoptysis were not independently associated with a poor outcome in the multivariate analysis (Table 5).

**Table 4:** Comparison of pulmonary actinomycosis patients with good and poor outcomes

	**Good outcome**	**Poor outcome**	***P *** **value**
	**(n = 59)**	**(n = 9)**	
Gender, male (%)	41 (69.5)	6 (66.7)	0.864
Mean age, years	59.6 ± 11.3	51.0 ± 11.3	0.038
Size of parenchymal lesion	3.7 (1.5-9.0)	5.2 (2.6-7.0)	0.093
Current smoker	17/56 (30.4)	3/8 (37.5)	0.697
Heavy alcoholics	7/52 (13.5)	2/9 (22.2)	0.609
Pulmonary comorbidity	21 (35.6)	4 (44.4)	0.608
Hemoptysis	32 (54.2)	8 (88.9)	0.071
Laboratory			
Normocytic normochromic anemia	25 (42.4)	5 (56.6)	0.458
White blood cell, ×10^3^/μL	7.9 (2.6–33.1)	8.0 (3.8–13.2)	0.246
CRP, mg/dL	1.5 (0.1–38.0)	4.3 (0.1–6.72)	0.726
Serum albumin, g/dL	3.9 (1.2–4.7)	3.8 (2.9–4.6)	0.450
Systemic disease			
Diabetes mellitus	11	1	1.000
Malignancy	6 (75.0)	2 (25.0)	0.335
1 month after initial antibiotic therapy			
Responder	38/41 (92.7)	2/9 (22.2)	< 0.001
Non responder	3/41 (7.3)	7/9 (77.8)	
Patients who did not use IV antibiotics	25 (42.4)	5 (55.6)	0.493
Antibiotics duration			
IV antibiotics, day	7 (2–21)	7 (5–8)	0.951
Initial therapy, months	5.3 (0.4-13.0)	10.2 (2.1–15.7)	0.061
With rescue therapy, months	5.3 (0.4–13.0)	13.2 (5.9–33.1)	< 0.001

**Table 5:** Clinical factors associated with a poor treatment outcome in patients with pulmonary actinomycosis

	**Univariate analysis**		**Multivariate analysis**	
	**P value**	**Crude OR (95% CI)**	**P value**	**Adjusted OR (95% CI)**
Age (continuous variable)	0.047	0.93 (0.86–0.99)	0.630	1.03 (0.93–1.13)
Male gender	0.864	0.89 (0.20–3.91)	0.609	1.84 (0.18–18.44)
Hemoptysis	0.080	6.75 (0.79–57.43)	0.305	3.97 (0.29–55.31)
Non response after 1 month	< 0.001	44.33 (6.23–315.50)	0.003	49.20 (3.34–724.30)

## Discussion

Our data suggest that treatment failure or recurrence of pulmonary actinomycosis is not uncommon. The 1-month antibiotic response was an independent predictor of the treatment outcome. This is the largest published series of biopsy-proven cases of pulmonary actinomycosis and is to our knowledge the first study to evaluate the treatment outcome using a multivariate analysis. The mean follow-up period of 37.7 months was also longer than in previous pulmonary actinomycosis studies.

In our series, the classic presentation of pulmonary actinomycosis with chest wall invasion and cutaneous fistulas discharging sulfur granules was absent. This was common in the pre-antibiotics era, but is now rare [3]. The change might be the result of improvements in oral hygiene, the ready availability of imaging modalities such as CT, and the early initiation of antibiotics when pulmonary infection is suspected [3]. A male predominance was noted and there were a large number of smokers and drinkers. These are consistent with the findings of earlier studies [6,11]. COPD and previous mycobacterial infection were the most common underlying respiratory disorders, and some patients had diabetes mellitus, as in previous studies [6,12]. However, it is not clear whether an immunocompromised status is a risk factor for actinomycosis. For example, the prevalence of actinomycosis was not increased in patients with acquired immunodeficiency syndrome [3]. In fact, the majority of patients in our study were immunocompetent. Education status also did not influence the incidence. A previous study also documented that socioeconomic class *per se* did not appear to correlate with the incidence of actinomycosis in the developed world [5].

The definitive diagnosis of actinomycosis needs the direct isolation of *Actinomyces* from a clinical specimen. However, cultures are positive in only a few cases because of failure of the anaerobic organism to grow [1]. The diagnosis is often based on histopathology showing Gram-positive filamentous branching bacteria and sulfur granules. All of our patients were diagnosed from biopsies. Microbiological cultures were performed for the biopsy specimens in 26 patients. However, the results were positive in only one patient.

Our treatment outcome for pulmonary actinomycosis consisted of a cure rate of 86.8% (59/68), failure rate of 8.8% (6/68), and relapse rate of 4.4% (3/68). There was no mortality from pulmonary actinomycosis. One patient died of metastatic gastric cancer 3 years after finishing successful treatment. Previously, our group reported 25 cases of pulmonary actinomycosis, and one patient died of a bronchopleural fistula (BPF) and empyema after a pneumonetomy [13]. Song et al. [6] reported an overall cure rate of 85% (34/40) in pulmonary actinomycosis without mentioning recurrence. They also experienced one case of postoperative mortality due to a BPF. Choi et al. [10] reported a 100% cure rate in 26 cases without recurrence, despite a relatively short follow-up duration (median 23 months). Possible explanations for treatment failure are drug-resistant co-pathogens and poor penetration of the drug caused by avascularity and induration of the infected area [14]. Although reported antimicrobial susceptibility tests indicate that most *Actinomyces* are susceptible to penicillin and amoxicillin, *Actinomyces* may acquire antibiotic resistance during antibiotic treatment [15,16].

There are no guidelines regarding the appropriate duration of antibiotic treatment. Recently, several investigators reported that shorter courses of treatment could be successful in pulmonary actinomycosis [10]. However, Kolditz et al. [12] reported that antibiotic treatment for less than 3 months in medically treated patients might be associated with local complications or recurrence. We managed cases of pulmonary actinomycosis with antibiotic treatment for less than 6 months. Furthermore, prolonged treatment did not prevent recurrence in three patients (range 9.2–14.0 months). It is not clear why these cases recurred: one was a current smoker and heavy drinker, but the other two had never smoked and did not consume alcohol. Considering the time to recurrence in our study, a reasonable follow-up duration should be at least 3 years.

Whether IV antibiotics should be used in the early management of actinomycosis remains uncertain. More than half of our medically treated patients were treated successfully without IV antibiotics. Furthermore, the median duration of IV antibiotics was less than 1 week in the group given IV antibiotics. Choi et al. [10] also reported that 7 of 15 medically treated patients with thoracic actinomycosis were treated successfully with oral antibiotics only. Therefore, the previous recommendation of IV antibiotics for 2–6 weeks might not be appropriate for all patients.

Penicillin G has been recommended as the drug of choice for treatment [2]. However, no comparative study has investigated which antibiotics are superior. To our knowledge, only two in vitro studies have reported *Actinomyces* drug susceptibility [15,16]. Recent reviews recommend that the first-line regimen consist of a beta-lactam and beta-lactamase inhibitor [1,16,17]. A beta-lactamase inhibitor offers the advantage of coverage against penicillin-resistant aerobic and anaerobic co-pathogens. These microorganisms produce the enzyme beta-lactamase, which can shield *Actinomyces* from the effect of penicillin. Furthermore, they might assist the spread of infection by inhibiting host defenses and reducing local oxygen tension [17]. In comparisons of AMX-CLV with AMP-SUL, the outcome of treatment with the latter tends to be poor. A possible explanation for this result is that sulbactam is a less potent beta-lactamase inhibitor than clavulanate in vitro [18], and ampicillin peak levels are delayed and lowered if it is ingested with food [19]. A prospective clinical trial would be the proper approach.

There are no definitive guidelines for when surgical intervention is required. Surgery is needed if a malignancy cannot be excluded [1]. It might also be used to control severe symptoms, such as uncontrolled hemoptysis [3]. Surgery could be a rescue treatment for patients who do not respond to antibiotics [6]. In our study, one of nine patients who presented with massive hemoptysis underwent emergency surgery. Bronchial artery embolization was attempted in the other eight patients, of whom seven ultimately needed surgery for continuing bleeding or for definitive diagnosis. Other indications for surgery include drainage of an abscess or pleural empyema, decortication, and the radical excision of sinus tracts [14].

One of our most important findings was that the 1-month antibiotic response and treatment outcome were closely associated according to the multivariate analysis. According to previous case reports, pulmonary actinomycosis usually decreases in size on the chest radiograph within 4 weeks [20]. A recent study also reported that only 20% (1/5) of patients with treatment failure showed any response to antibiotics within 4 weeks [6]. In addition, long-term antibiotics could not guarantee a successful outcome or no relapse in our series. The absence of an early radiological response might be an important predictor of a poor outcome. We did not find a significant difference in treatment outcomes based on treatment modality.

This study had several limitations. First, given it's retrospective nature, selection bias may have influenced the significance of our results. The sample size of the study was too small to obtain a strong conclusion. Additionally, many treatment options were used, and this might have influenced the statistical power. Second, there were no predetermined criteria for halting treatment. Antibiotics were stopped in most patients based on radiological and clinical resolution of their pulmonary actinomycosis. Third, we did not evaluate the correlation between drug susceptibility test results and treatment outcome.

## Conclusion

In conclusion, antibiotic treatment with or without surgery was effective for treatment of pulmonary actinomycosis. The duration of treatment for pulmonary actinomycosis can be shorter than traditionally recommended. Nevertheless, treatment failure and recurrence are not uncommon. Therefore, close monitoring and follow-up are necessary for a successful outcome. In addition, the early antibiotic response can predict treatment outcome, and might have a role in patient follow-up.

## Competing interests

The authors declare that they have no competing interests.

## Authors’ contributions

JYP: drafting the manuscript, enrolling subjects, data collection, and data analysis. TL, HL, HJL, JL: data collection and data analysis. JSP, YJC, YSP, CHL: analysis and interpretation of data. SML, HIY, JJY: read the CT scans, interpretation of data. CGY, YWK, SKH, CTL: contributed to the study concept and design. JHL: contributed to the study concept and design; acquisition, analysis, and interpretation of the data; drafting of the manuscript for important intellectual content. All authors contributed substantially to drafting and editing the manuscript and approval of the final version to be published.

## Pre-publication history

The pre-publication history for this paper can be accessed here:

http://www.biomedcentral.com/1471-2334/14/10/prepub
